# Establishing resilience-targeted prediction models of rainfall for transportation infrastructures for three demonstration regions in China

**DOI:** 10.1038/s41598-024-66249-w

**Published:** 2024-07-05

**Authors:** Wen Zeng, Xiaodan Sun, Hongping Xing, Yu Liu, Lu Liu

**Affiliations:** 1 School of Civil Engineering, Southwest Jiaotong University, Chengdu, 610031 China; 2 National Engineering Research Center of Geological Disaster Prevention Technology in Land Transportation, Chengdu, 610031 China; 3 MOE Key Laboratory of High-Speed Railway Engineering, Southwest Jiaotong University, Chengdu, 610031 China

**Keywords:** Projection and prediction, Natural hazards

## Abstract

Rainstorm is one of the global meteorological disasters that threaten the safety of transportation infrastructure and the connectivity of transportation system. Aiming to support the resilience assessment of transportation infrastructure in three representative regions: Sichuan–Chongqing, Yangtze River Delta, and Beijing-Tianjin-Hebei-Shandong, rainfall data over 40 years in the three regions are collected, and the temporal distribution of rainfall are analyzed. Prediction equations of rainfall are established. For the purpose of this, the probabilistic density function (PDF) is assigned to the rainfall by fitting the frequency distribution histogram. Using the assigned PDF, the rainfall data are transformed into standard normal space where regression of prediction equations is performed and the prediction accuracy is tested. The results show that: (1) The frequency of rainfall in the three regions follows a lognormal distribution based on which the prediction equations of rainfall can be established in standard normal space. The error of regression shows no remarkable dependence on self-variables, and the significance analysis indicates that the equations proposed in this paper are plausible for predicting rainfalls for the three regions. (2) The Yangtze River Delta region has a higher risk of rainstorm disaster compared to the other two regions according to the frequency of rainfall and the return period of precipitation concentration. (3) Over the period of 1980–2021, the Sichuan–Chongqing region witnessed an increase in yearly rainfall but a decrease in rainstorm disasters, whereas the other two regions experienced a consistent rise in both metrics.

## Introduction

**Figure 1:**
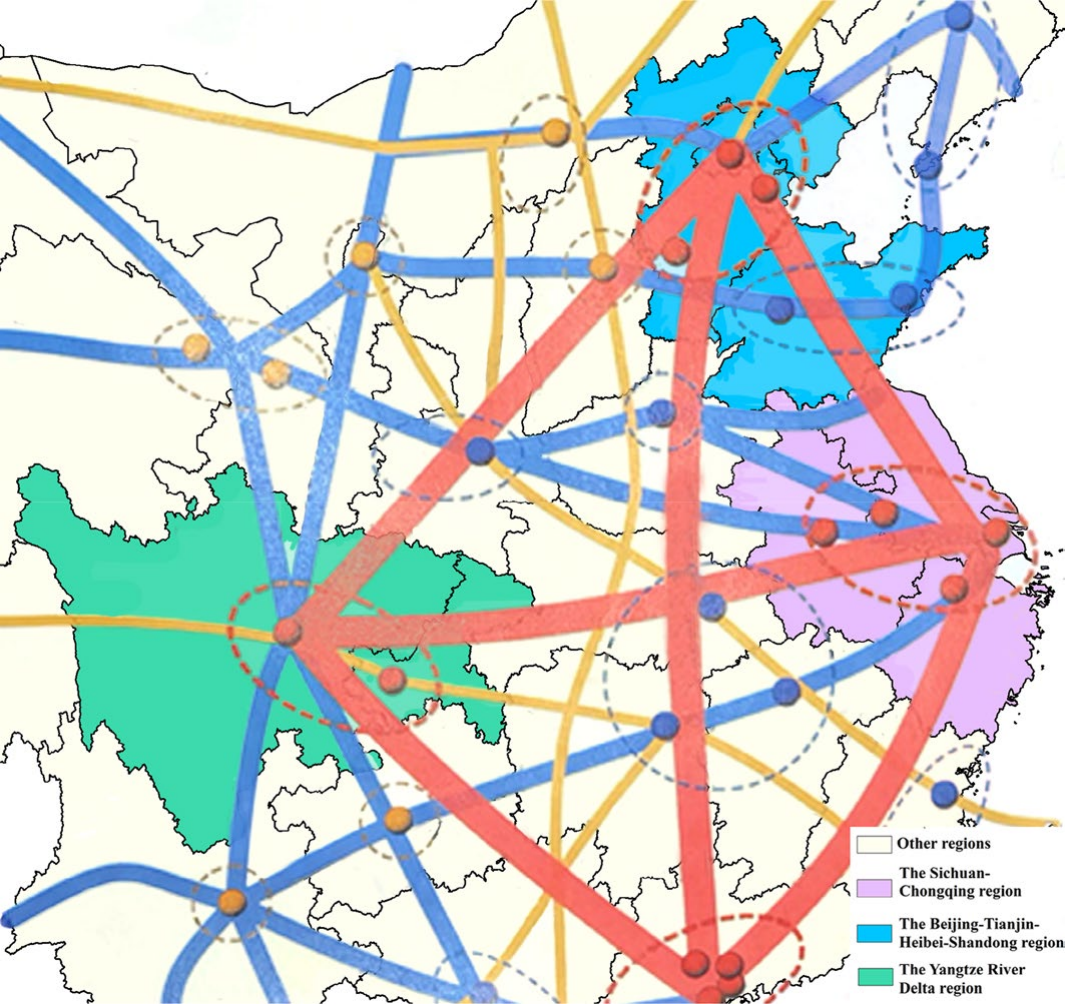
Schematic layout of the main skeleton of the national comprehensive three-dimensional transportation network (source of the base map: Outline of the National Comprehensive Three-Dimensional Transportation Network Plan—Annex).

The "National Comprehensive Stereo Transportation Network Planning Outline," published in February 2021, highlights the imperative to enhance China's transportation network resilience. The evolving domestic and international landscape has presented fresh, more demanding imperatives for expediting the development of a strong transportation country and establishing a modern, high-quality, three-dimensional national transportation network. The Sichuan–Chongqing region, Beijing-Tianjin-Hebei-Shandong region, and the Yangtze River Delta region are three typical regions in China with geographic locations and geological and climatic environments (Fig. 1). Furthermore, these regions cover China's political and economic hubs, underscoring the paramount significance of safeguarding the safety of the transportation infrastructure within them. Specifically, the Beijing-Tianjin-Hebei-Shandong region features a terrain that inclines from northwest to southeast, transitioning from mountainous highlands to plains and hilly terrain as one moves from northwest to southeast^1^. This region falls within the temperate continental monsoon climate zone, characterized by scorching, rainy summers and frigid, arid winters. The influence of monsoons results in recurrent droughts and floods. Encompassing 41 cities, including Shanghai, as well as provinces like Jiangsu and Zhejiang, the Yangtze River Delta region is situated in the lower Yangtze River basin, bounded by the Yellow Sea and the East China Sea. It experiences a predominantly subtropical monsoon climate characterized by warm, sweltering summers and abundant rainfall. Given its high population density and unique economic status, the region suffers from a high impact of disaster losses^2,3^. Serving as the political and economic epicenter of southwestern China, the Sichuan–Chongqing region is located in the transition zone from the Qinghai-Tibetan Plateau to the plains of the middle and lower Yangtze River basin. To the east lies the basin, while the west comprises the western Sichuan Plateau and the southern Sichuan Mountains. This geographical diversity results in significant climate variations across the region^4^. Transportation infrastructures in these regions assume an important role in connectivity and support economic development, regional coordination, and improvement of people's livelihood^5^. It is of great significance for ensuring the operational safety of transportation infrastructure to estimate the disaster risk of transportation infrastructure in the region and explore the mechanism of disaster impact on an urban scale.

China is one of the countries with the highest frequency of rainstorms and flooding in the world, and the proportion of disasters caused by rainstorms in disaster-causing meteorological events in China is as high as 40%^6^. In 2012, Beijing experienced a severe rainstorm on July 21st, leading to the inundation of 95 urban roads due to waterlogging, essentially paralyzing ground transportation and resulting in 79 casualties^7^. In late July 2023, the Beijing region encountered its most substantial precipitation in 140 years, driven by the impact of Typhoon "Dusu Rui." Between 20:00 on July 29th and 13:00 on July 31st, 2023, the Beijing area witnessed an average rainfall of 176.9 mm, with the Mentougou Alpine Rose Garden experiencing the most intense precipitation at 580.9 mm^8^. This excessive rainfall led to 11 fatalities and left 27 individuals reported missing^9^. In terms of hydraulic projects, more than 110 rivers experienced excessive flooding, and more than 280 km of river embankments were damaged; 4 medium-sized reservoirs, 13 small reservoirs, and 16 sluices suffered varying degrees of damage^10^. The Yangtze River Delta is another region seriously affected by typhoons and threatened by heavy rainfall, with losses of 178.96 billion yuan due to rainstorm disasters in the middle and lower reaches of the Yangtze River in 2020 alone^11^. The Sichuan–Chongqing region, while less prone to typhoons, faces the risk of heavy summer rainfall-induced secondary disasters like landslides and mudslides due to its elevated terrain and intricate topography, posing a serious threat to transportation routes in mountainous areas. From the night of August 12th to the early morning of August 13th, 2010, heavy localized rainfall struck Qingping Township in Mianzhu City, Sichuan Province. This downpour triggered concurrent mudslides in eleven nearby ravines, resulting in the loss of 14 lives, including fatalities and missing persons, and the destruction of 379 houses. Simultaneously, the Hanqing Highway suffered interruptions, and two bridges were obliterated^12^. On June 4th, 2022, the D2809 passenger train encountered a mudslide that abruptly descended onto the railway track while approaching the entrance of the Yuezhai Tunnel, situated in front of Rongjiang Station on the Guiguang Line. This event led to the derailment of two train carriages^13^. Presently, research on rainstorm disasters in the Beijing-Tianjin-Hebei-Shandong, Yangtze River Delta, and Sichuan–Chongqing regions has primarily focused on analyzing individual cases of heavy rainfall events or extreme precipitation processes^14–16^. However, there is also a growing understanding of the temporal distribution characteristics of heavy rainfall disasters. For instance, rainstorm in Southwest China mainly occurs in the warm season, with rainfall amounts typically ranging from 100 to 300 mm and rainstorm days averaging around 1 to 4 days. The interannual variation of precipitation and precipitation days is consistent. Nevertheless, over the past 50 years, Southwest China has witnessed an escalation in both the quantity and frequency of rainstorm events, accompanied by extreme intensity levels^17–20^. In the Beijing-Tianjin-Hebei-Shandong region, rainstorms exhibit relative concentration in late July and early August, with a multi-year average of 1.3 rainstorm days across the entire area. Since 1961, there has been a decreasing trend in the precipitation, days and intensity of rainstorms in most regions of Beijing-Tianjin-Hebei. After the 2000s, the amount of rainstorms further decreased^21–23^. In the middle and lower reaches of the Yangtze River, rainstorm events primarily span 2–3 days, with a maximum duration of 8 days. These events predominantly manifest during the summer months, particularly in June and July. Rainstorm events in this area exhibit both long-term trends and inter-decadal variations, with a noteworthy increase in occurrence frequency over the past 58 years. Furthermore, heavy and extremely heavy rainstorms have displayed a significant uptick in all seasons over the last decade^24–26^.

The ultimate goal of disaster research is to provide support and reference for pre-disaster prediction and post-disaster rapid response to regional transportation infrastructure risks. Due to the complexity of the disaster formation mechanism and the limited number of disasters in rainstorms, the methods and accuracy of pre-disaster prediction have been a focal point of attention. Wang Xiuying et al.^27^ established a short-term heavy precipitation forecasting model for Pu'er City based on a multiple linear regression model. Their findings demonstrated the model's robust predictive capabilities, rendering it suitable for short-term heavy precipitation forecasting and alerts in Pu'er. Milan Gocic et al.^28^ employed linear regression to forecast the precipitation trend using monthly precipitation data from 29 stations spanning the period from 1946 to 2012. Their findings indicated a consistent upward trend in annual precipitation levels in Serbia throughout this timeframe. He Xinguang et al.^29^ developed a rainfall forecasting model using monthly historical rainfall data and climate indices by incorporating the multi-resolution analysis (MRA) and multiple linear regression (MLR) model. Their findings revealed that the proposed MRA-based model provides considerably more accurate monthly rainfall forecasts for all of the selected stations over South Australia than the traditional regression model. S.K.Chandniha et al^30^ advocated the statistical downscaling model (SDSM) that is based on the multiple linear regression (MLR) technique to assess the likely future monthly rainfall in Piperiya watershed of Chhattisgarh state in India. The results showed it will help in studying the effect of climate change on the expected rainfall in this particular area. In recent years, a variety of prediction models, including Markov models, grey system models, spectral analysis models, and others, have been gradually used for rainstorm disaster prediction^31–33^. Sun Caizhi et al.^34^ used precipitation data from a hydrological station in Shanxi Province for the past 50 years as an illustrative case. They utilized a weighted Markov chain model to forecast the variability of abundance and dried-up of precipitation in the future and achieved favorable outcomes. Feng Lihua^35^ used the previous period's forecast factors for calculation and endowed grey clustering with the function of analysis and prediction, applying it to predicting and analyzing the precipitation trends. Li Ping et al.^36^ used the precipitation data of Caizuizi Station in the Naolihe River Basin from 1964 to 2003 as an example and used spectral analysis for forecasting and analysis. The results indicated there are two main cyclicalities of annual precipitation in the area (about three years and nine years), reflecting the climate change patterns in the area. Furthermore, with the continuous development of computer technology, machine learning algorithms have been gradually applied to precipitation forecasting, especially the neural network model, which has been widely used^37,38^. Gou Zhijing et al.^39^ established a genetic neural network prediction model based on the daily surface climate data from 13 stations in Tianjin from 1951 to 2006, and then conducted an experiment using rainfall level as the decision-making attribute. Their findings indicated that the prediction accuracy of this method for all precipitation levels is better than the traditional neural network algorithm. Liu Yang et al.^40^ used a multi-hidden layer neural network to establish the nonlinear relationship between rainfall and various parameters and to forecast rainfall in the short term. The experimental results showed that the prediction model based on a multi-hidden layer neural network can predict more than 95% of the rainfall events, and the misreporting rate is only about 20%.

In contrast to accurate mathematical analysis based on a large-scale regional data volume, the demand for disaster emergency response in regional transportation infrastructure networks is more reflected in convenience for predicting the scope and intensity of disaster impact. Based on the need for resilience assessment of transportation infrastructure networks in the Beijing-Tianjin-Hebei-Shandong, Yangtze River Delta, and the Sichuan–Chongqing regions, this paper collects and statistically analyzes about 40 years of precipitation and climate change data. It aims to study the temporal distribution characteristics of rainfall in these three regions, along with the characteristic parameters of rainstorm disasters. Subsequently, a rainstorm disaster prediction model is developed based on these research findings.

## Data

Daily and annual rainfall data spanning 42 years have been collected for the three representative demonstration areas: Sichuan–Chongqing, Beijing-Tianjin-Hebei-Shandong, and the Yangtze River Delta, which are used for analyzing the monthly and annual distribution of rainfall separately, as presented in Table 1. Specifically, data encompassing daily and annual rainfall from 30 meteorological stations in the Sichuan–Chongqing region, 34 meteorological stations in the Beijing-Tianjin-Hebei-Shandong region and 30 meteorological stations in the Yangtze River Delta region are chosen, aggregating to a sum of 94 meteorological stations, with the time series from 1980 to 2021. Traditional studies in rainstorm climatology predominantly concentrate on rainfall exceeding 50 mm without further delineation. By considering 24-h rainfall, rainstorms can be further categorized as heavy or extremely heavy. In this study, rainfall classifications adhere to the criteria outlined in "the standard of rainfall level," wherein a rainstorm is characterized by 24-h rainfall equal to or exceeding 50 mm and less than 100 mm, a heavy rainstorm by 24-h rainfall equal to or exceeding 100 mm and less than 250 mm, and an extremely heavy rainstorm by 24-h rainfall equal to or exceeding 250 mm^41^. Table 2 displays the chosen rainstorm characteristic variables, facilitating the analysis of temporal rainfall variation patterns across the three region.

**Table 1:** Details of rainfall data.

Region	Number of weather stations	Details of information	Time series	Source
Sichuan–Chongqing	30	Daily and yearly rainfall data (including station latitude and longitude, daily rainfall, annual rainfall, etc.)	From1980 to 2021	Wheat software (http://www.wheata.cn/)
Beijing–Tianjin–Hebei–Shandong	34			
Yangtze River Delta	30

**Table 2:** Characteristic variables of rainfall.

Variables	Definition	Unit
Rainstorms	50 mm ≤ *R*_24_ < 100 mm	mm
Heavy rainstorms	100 mm ≤ *R*_24_ < 250 mm	mm
Extremely heavy rainstorms	*R*_24_ ≥ 250 mm	mm
Rainstorm days	Total number of rainstorm days at all meteorological observation stations in the study area during the study period	day
Annual rainfall	Total annual rainfall for all meteorological observation stations in the study area	mm

Where *R*_24_ represents the rainfall within 24 h.

**Figure 2:**
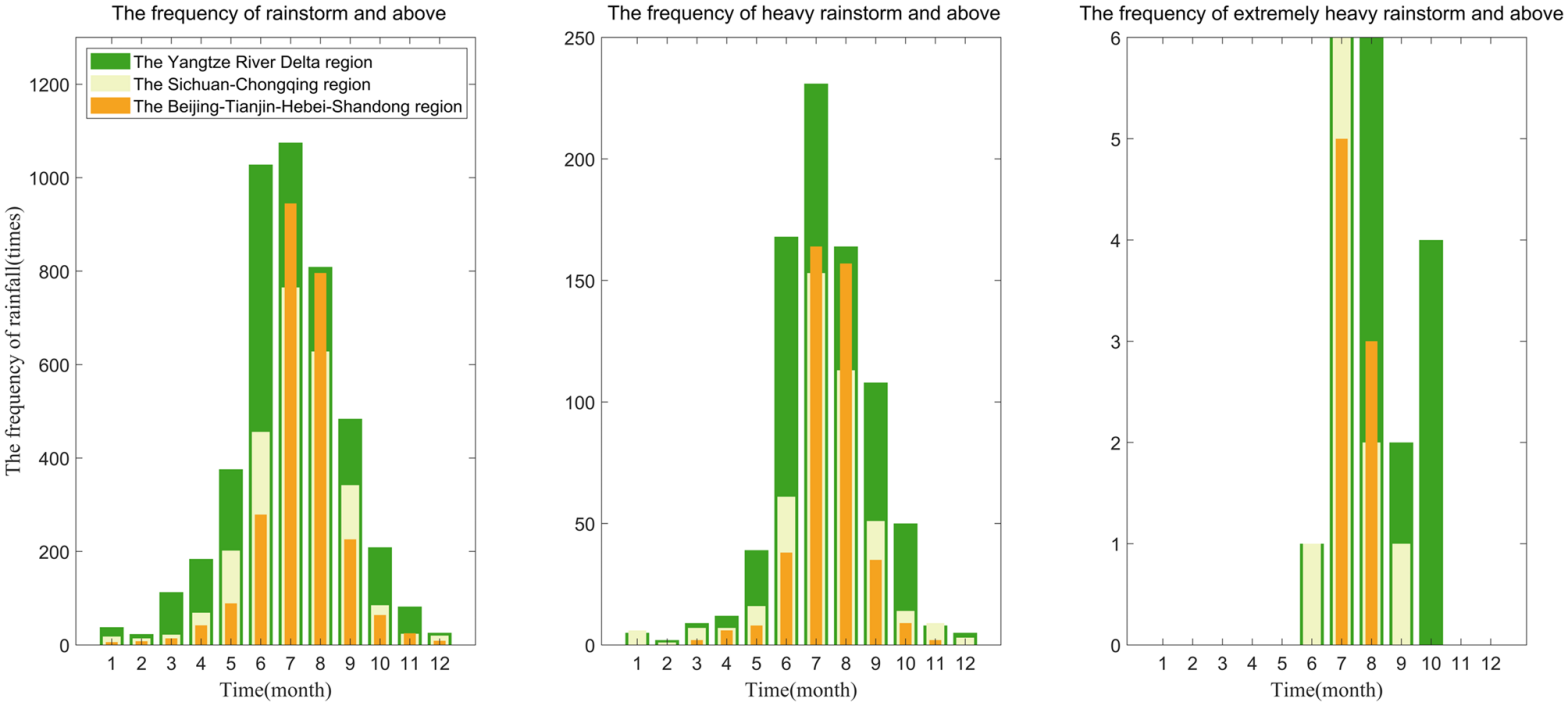
Monthly distribution of the frequency of rainstorms/heavy rainstorms/extremely heavy rainstorms and above in three districts.

Figure 2 illustrates the monthly distribution of the frequency of rainstorms, heavy rainstorms, extremely heavy rainstorms and above in the three demonstration areas. As seen in Fig. 2, the peak monthly occurrence of rainstorms, heavy rainstorms, and extremely heavy rainstorm disasters in the three demonstration areas occurs in July. Rainstorm disasters are predominantly concentrated in the Sichuan–Chongqing region from May to September, with heavy rainstorm disasters being particularly prominent from June to September. In the Beijing-Tianjin-Hebei-Shandong region, rainstorms and heavy rainstorm disasters primarily occur from June to September. In contrast, the Yangtze River Delta region experiences a more extended concentration period of rainstorms and heavy rainstorm disasters, typically spanning from May to October. The monthly distribution of extremely heavy rainstorm disasters in the three demonstration areas no longer has regularity, only occurring from June to October. Moreover, the monthly occurrence of rainstorms, heavy rainstorms, and extremely heavy rainstorm disasters is typically higher in the Yangtze River Delta region compared to the other two regions. During July and August, the Beijing-Tianjin-Hebei-Shandong region experiences a higher incidence of rainstorms and heavy rainstorm disasters compared to the Sichuan–Chongqing region.

**Figure 3:**
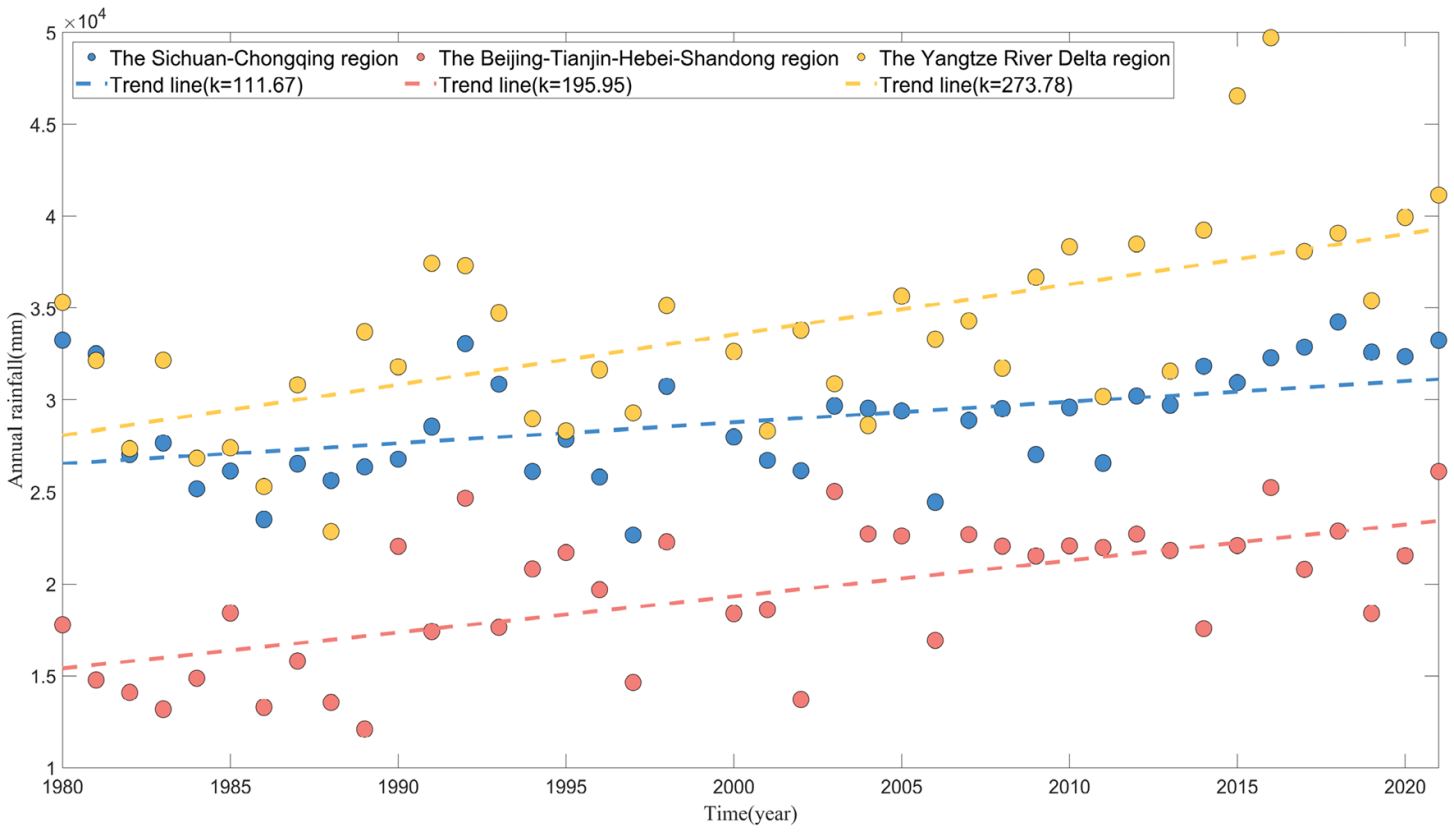
Temporal distribution of yearly rainfall in three regions.

**Figure 4:**
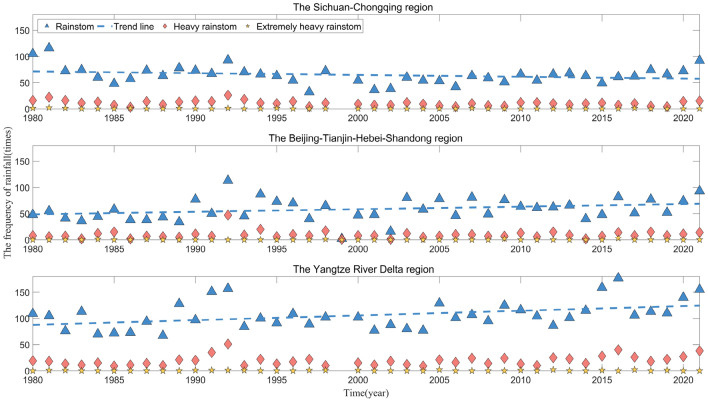
Annual distribution of the frequency of rainstorms/heavy rainstorms/extremely heavy rainstorms and above in three districts.

Figure 3 illustrates the temporal distribution of annual rainfall in the three demonstration areas, revealing significant interannual fluctuations and variations. A comparison of the three demonstration areas shows that the yearly rainfall in the Yangtze River Delta region is much higher than the other two areas from 1980 to 2021, and the yearly rainfall in the Sichuan -Chongqing region is more prominent than that in the Beijing-Tianjin-Hebei-Shandong region. In addition, the trend lines of the three demonstration areas are all a gradually increasing straight line, with slopes of 111.67, 195.95, and 273.78 in the Sichuan–Chongqing, Beijing-Tianjin-Hebei-Shandong, and Yangtze River Delta regions, respectively. This indicates that the annual rainfall increases at the fastest rate in the Yangtze River Delta region, followed by the Beijing-Tianjin-Hebei-Shandong region, and the slowest in the Sichuan–Chongqing region in the period from 1980 to 2021. Figure 4 further demonstrates the annual distribution of the frequency of rainstorms, heavy rainstorms, and extremely heavy rainstorms and above in the three demonstration areas. A comparison of the trend lines reveals that, in line with the annual rainfall distribution characteristics, the frequency of rainstorm disasters in the Yangtze River Delta and Beijing-Tianjin-Hebei-Shandong regions continues to exhibit a year-by-year increase from 1980 to 2021. Notably, the Yangtze River Delta region (with a slope of 0.9004) demonstrates a faster rate of increase than the Beijing-Tianjin-Hebei-Shandong region (with a slope of 0.4928). However, the frequency of rainstorm disasters in the Sichuan -Chongqing region shows an opposite trend to the yearly rainfall.

## Methodology

The process of forming the rainfall prediction models is:

Step 1. Assign a specific probability distribution type to each dataset based on the frequency distribution histogram of the original rainfall data in the three demonstration areas. Moreover, we need to perform a goodness-of-fit test on the specified probability density function by means of the chi2gof test. The equation is as follows:

1 $${x}^{2}=\sum_{i=1}^{N}\frac{{\left({O}_{i}-{E}_{i}\right)}^{2}}{{E}_{i}}$$

where ${O}_{i}$ are the observed counts and ${E}_{i}$ are the expected counts based on the hypothesized distribution.

Step 2. Transform the original rainfall data into standard normal space using the assigned PDF to meet the assumption of normal distribution of multiple linear regression. Once the suitable probability distribution is determined for each demonstration area, the sample values of the original data can be standardized using Eq. (2).

2 $${v}_{i}={\Phi }^{-1}\left[{F}_{{\theta }_{i}}\left({\theta }_{i}\right)\right]\text{ i}=\text{1,2},3$$

where ${\theta }_{i}$ denotes data from three distinct regions: i = 1 for the Sichuan–Chongqing region, i = 2 for the Beijing-Tianjin-Hebei-Shandong region, and i = 3 for the Yangtze River Delta region. ${F}_{{\theta }_{i}}\left({\theta }_{i}\right)$ represents the edge cumulative probability distribution density function fitted by the data of ${\theta }_{i}$, while ${\Phi }^{-1}[]$ stands for the inverse function of the standard normal cumulative distribution function. The variable ${v}_{i}$ comprises a series of standard normal random variables. Equation (2) facilitates the transformation of ${\theta }_{i}$ data into the ${v}_{i}$ format, and utilizes ${v}_{i}$ to perform multiple linear regression in standard normal space, which ultimately lays a good foundation for establishing the prediction equations for the converted data^42^.

Step 3. Select three input variables for models in the three demonstration areas using correlation analysis. The prediction models established in this paper will be used as the basis for predicting and evaluating rainfall in the three demonstration zones. In order to improve the practicability of the model, specific criteria have been established for selecting variables: the input variables should not be more than three; the data for these variables should be readily accessible and operationally feasible to collect; and the chosen variables should be representative of the general pattern of rainfall.

In statistics, the correlation between variables can be measured by a statistical value, the correlation coefficient. The correlation coefficient generally takes values in the range of [-1,1]. If there is a linear relationship between two variables, the correlation coefficient is positive; if there is a negative linear relationship between the two variables, the correlation coefficient is a negative number. Additionally, the closer the absolute value of the correlation coefficient is to 1, the stronger the correlation between the two variables. In contrast, the closer it is to 0, the weaker the correlation.

Step 4. Establish the prediction models and assess the performance of established models using various inspection methods based on the SPSS software. In standard normal space, we use the theory of multiple linear regression to construct rainfall prediction models for three demonstration areas based on the SPSS software. Three key assumptions are made in this approach: Assumption 1: The selected samples are independent of each other; Assumption 2: The samples exhibit no covariance; Assumption 3: The residuals follow a normal distribution^43^.

Under three assumptions, a prediction model containing three independent variables is established using multiple linear regression, which can be represented as follows:

3 $$\text{y}={a}_{0}+{a}_{1}{x}_{1}+{a}_{2}{x}_{2}+{a}_{3}{x}_{3}+\varepsilon $$

In the equation, ${a}_{0},{a}_{1},{a}_{2},\dots ,{a}_{n}$ represent regression coefficients and ε denotes random error.

## Probability distribution assignment

**Figure 5:**
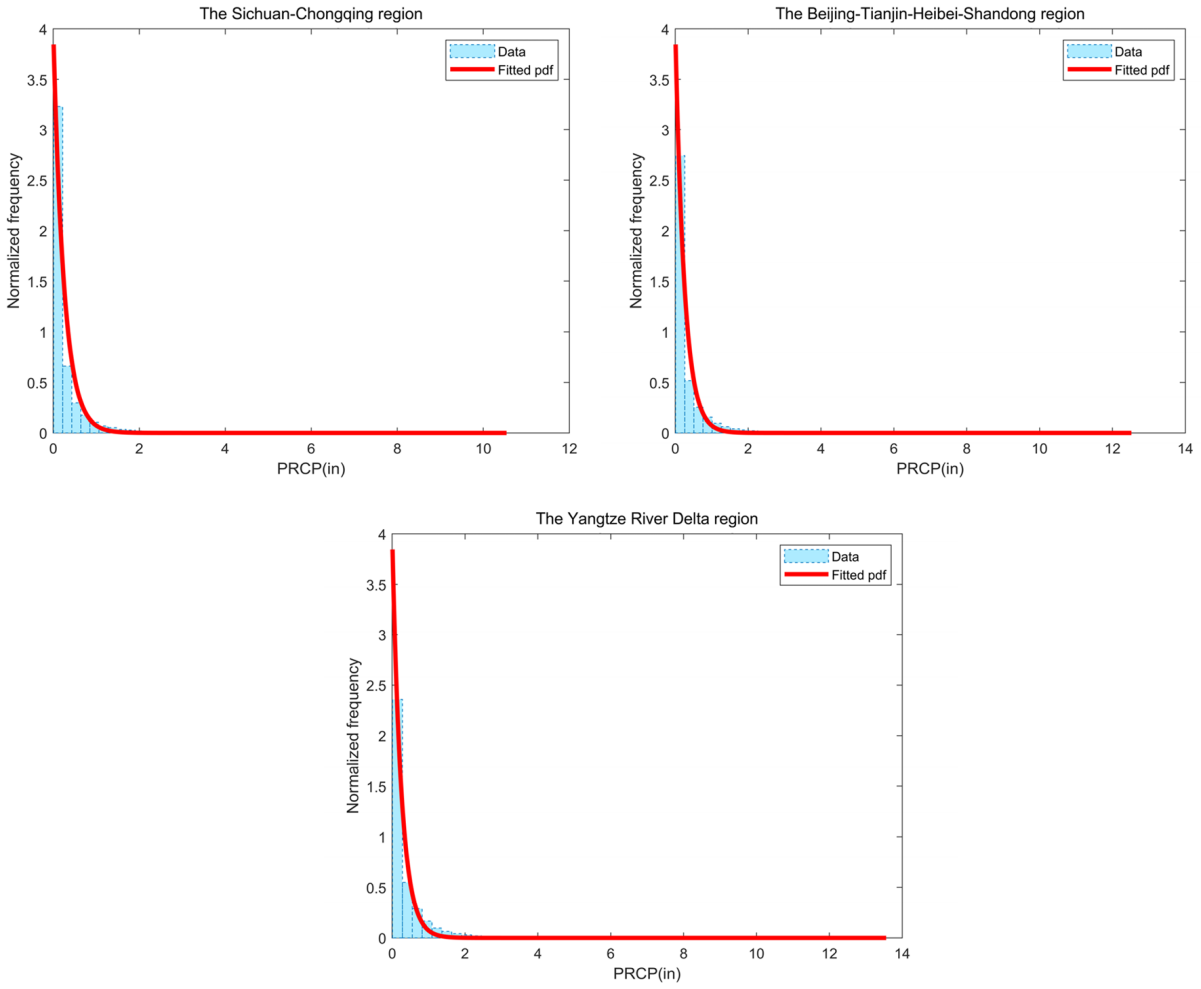
Histograms of rainfall frequency and probability density function curves for the three regions.

**Figure 6:**
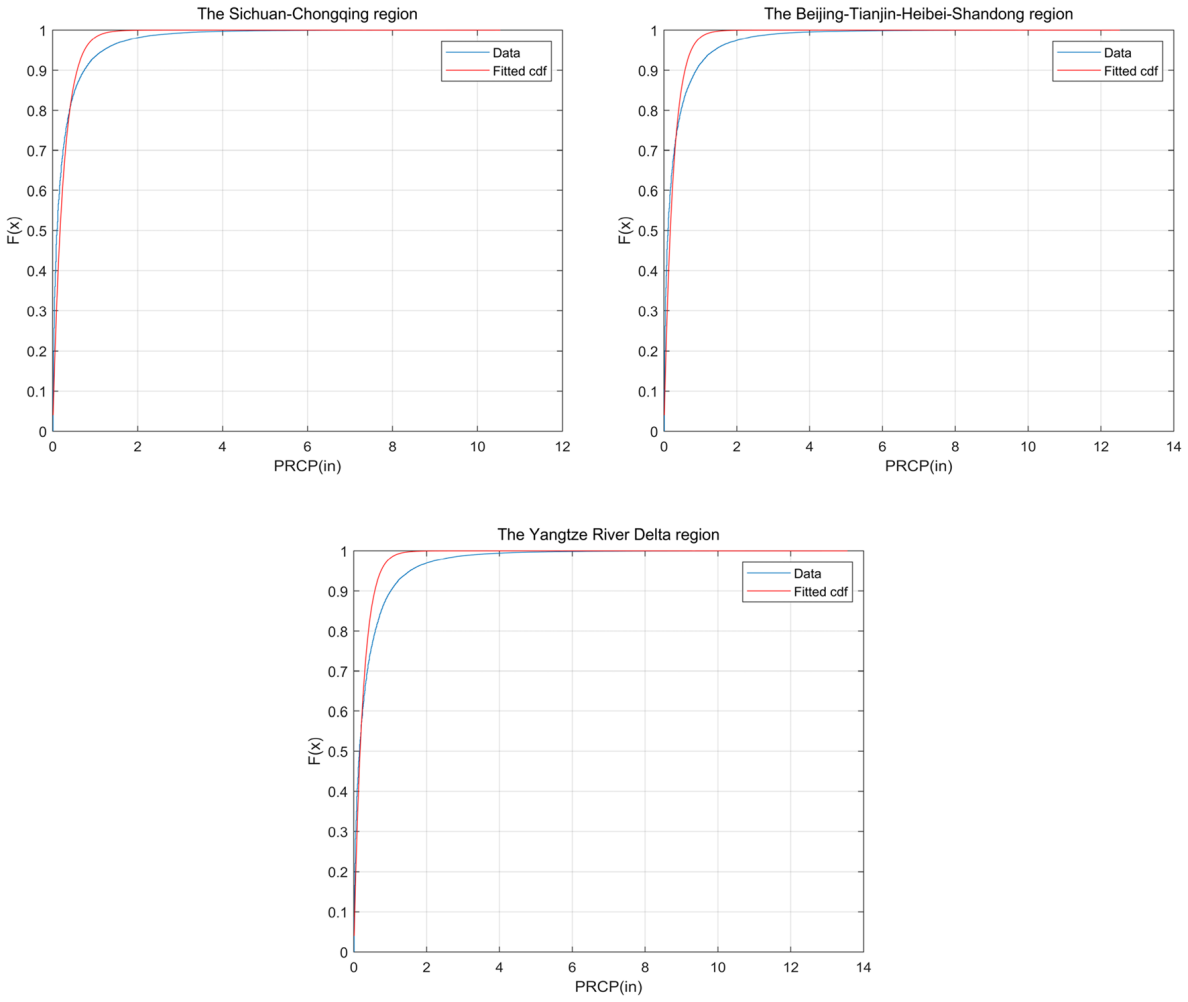
Cumulative probability function curves for the three regions.

Prior to regression analysis, it is necessary to consider data transformation if the data do not adhere to a normal distribution. Using rainfall data from the three demonstration areas, we have plotted frequency histograms of the raw data, as depicted in Fig. 5. The figure reveals that the distribution characteristics of the original rainfall data in the three demonstration areas exhibit similarity, displaying highly uneven distribution with a predominant concentration below 0.5 inches. At the same time, there are differences in the distribution characteristics of raw rainfall data in the three demonstration areas.The low-frequency component of rainfall is the highest in the Sichuan–Chongqing region, followed by the Beijing–Tianjin–Hebei–Shandong region, and the lowest in the Yangtze River Delta region, indicating that rainfall in the Sichuan–Chongqing region is heavily dominated by drizzle. However, the relationship between the maximum rainfall in the three regions is exactly the opposite. Data conforming to a normal distribution is a prerequisite for regression analysis, and assigning a probability distribution to the raw data for each region aids in the conversion of the data to standard normal space. Consequently, we assign a specific probability distribution type to each dataset, which lays the foundation for transforming the data into the standard normal space. Table 3 presents a summary of parameter statistics for the three demonstration areas. Notably, the raw rainfall data in the Sichuan–Chongqing, Beijing-Tianjin-Hebei-Shandong, and Yangtze River Delta demonstration areas all exhibit conformity to the single-sided exponential distribution. Probability density function curves and cumulative probability distribution curves are drafted in Figs. 5 and 6, respectively, indicating a close alignment between the original data from the three regions and their respective designated probability distribution types. According to the result of the chi2gof test, the test values are all 0, which shows that chi2gof does not reject the null hypothesis at the default 5% significance level and there is a good fit between the data and the theoretical distribution.

**Table 3:** Statistical summary of parameter for the three regions.

Region	Min	Max	Average	Standard deviation	Fit distribution	Range
Sichuan–Chongqing	0.01	10.54	0.2902	0.5340	Single-sided exponential	[0,12]
Beijing–Tianjin–Hebei–Shandong	0.01	12.52	0.3348	0.6247	Single-sided exponential	[0,14]
Yangtze River Delta	0.01	13.56	0.3951	0.6748	Single-sided exponential	[0,14]

Formulas for the probability density function curve and cumulative probability distribution curve for the single-sided exponential distribution are provided in Eqs. (4) and (5), respectively.

4 $${f}_{x}\left(i\right)=b*\text{exp}(-a*x\left(i\right))$$

5 $${F}_{x}\left(i\right)=-(b/a)*(\text{exp}\left(-a*x\left(i\right)\right)-1)$$

In the equation, we determined two parameters by fitting the data, so we have $a=b=4$, $x\left(i\right)$ represents a specific data point.

## Correlation analysis

For the purpose of assessing the correlation between the input variables and rainfall, this section conducted a correlation analysis on each input variable, as shown in Tables 4, 5 and 6. Taking the Sichuan–Chongqing region as an example, the Pearson correlation coefficients among the variables within the rainfall system are presented in Table 4. The table encompasses not only the dependent variable, rainfall ${P}_{RCP}$, but also the independent variables involved, including mean temperature ${T}_{EMP}$, dew point temperature ${D}_{EWP}$, sea level pressure ${S}_{LP}$, station pressure ${S}_{TP}$, mean wind speed ${W}_{DSP}$, maximum sustained wind speed ${M}_{XSPD}$, maximum air temperature ${M}_{AX}$, and minimum air temperature ${M}_{IN}$. From Table 4, it can be seen that the correlation coefficient between ${P}_{RCP}$ and ${D}_{EWP}$ is 0.305, which shows a positive correlation between the two variables. However, the correlation between ${D}_{EWP}$ and ${T}_{EMP}$, ${M}_{AX}$, and ${M}_{IN}$ is 0.950, 0.854, and 0.977, respectively, indicating that there is a strong correlation between ${D}_{EWP}$ and these three variables, making them unsuitable for the same model. Considering the input parameter criteria, we apply a similar approach to determine the other two input variables, ${S}_{LP}$ an ${W}_{DSP}$, which exhibit correlation coefficients of -0.293 and 0.119 with ${P}_{RCP}$. Although not significant, it still indicates a certain correlation between them. Therefore, the input variables of the rainfall prediction model in the Sichuan–Chongqing region are determined as dew point temperature ${D}_{EWP}$, sea level pressure ${S}_{LP}$, and mean wind speed ${W}_{DSP}$. Similarly, the input variables of the remaining two regions can be determined as dew point temperature ${D}_{EWP}$, sea level pressure ${S}_{LP}$, and mean wind speed ${W}_{DSP}$ for the Beijing-Tianjin-Hebei-Shandong region, while mean temperature ${T}_{EMP}$, level pressure ${S}_{LP}$, maximum sustained wind speed ${M}_{XSPD}$ for the Yangtze River Delta region.

**Table 4:** Results of correlation analysis of rainfall system parameters in the Sichuan–Chongqing region.

	${P}_{RCP}$	${T}_{EMP}$	${D}_{EWP}$	${S}_{LP}$	${S}_{TP}$	${W}_{DSP}$	${M}_{XSPD}$	${M}_{AX}$	${M}_{IN}$
${P}_{RCP}$	1.000	0.245	0.305	−0.293	−0.006	0.119	0.114	0.244	0.269
${T}_{EMP}$	0.245	1.000	0.950	−0.742	0.271	−0.041	−0.021	0.952	0.967
${D}_{EWP}$	0.305	0.950	1.000	−0.662	0.350	−0.100	−0.086	0.856	0.977
${S}_{LP}$	−0.293	−0.742	−0.662	1.000	0.177	−0.128	−0.139	−0.795	−0.662
${S}_{TP}$	−0.006	0.271	0.350	0.177	1.000	−0.190	−0.192	0.110	0.363
${W}_{DSP}$	0.119	−0.041	−0.100	−0.128	−0.190	1.000	0.827	0.045	−0.092
${M}_{XSPD}$	0.114	−0.021	−0.086	−0.139	−0.192	0.827	1.000	0.066	−0.081
${M}_{AX}$	0.244	0.952	0.856	−0.795	0.110	0.045	0.066	1.000	0.871
${M}_{IN}$	0.269	0.967	0.977	−0.662	0.363	−0.092	−0.081	0.871	1.000

**Table 5:** Results of correlation analysis of rainfall system parameters in the Beijing–Tianjin–Hebei–Shandong region.

	${P}_{RCP}$	${T}_{EMP}$	${D}_{EWP}$	${S}_{LP}$	${S}_{TP}$	${W}_{DSP}$	${M}_{XSPD}$	${M}_{AX}$	${M}_{IN}$
${P}_{RCP}$	1.000	0.229	0.315	−0.270	0.102	0.036	0.030	0.216	0.267
${T}_{EMP}$	0.229	1.000	0.940	−0.841	0.220	−0.198	−0.180	0.972	0.974
${D}_{EWP}$	0.315	0.940	1.000	−0.803	0.165	−0.191	−0.192	0.885	0.964
${S}_{LP}$	−0.270	−0.841	−0.803	1.000	−0.378	0.095	0.063	−0.831	−0.819
${S}_{TP}$	0.102	0.220	0.165	−0.378	1.000	−0.179	−0.124	0.263	0.177
${W}_{DSP}$	0.036	−0.198	−0.191	0.095	−0.179	1.000	0.903	−0.215	−0.162
${M}_{XSPD}$	0.030	−0.180	−0.192	0.063	−0.124	0.903	1.000	−0.182	−0.163
${M}_{AX}$	0.216	0.972	0.885	−0.831	0.263	−0.215	−0.182	1.000	0.921
${M}_{IN}$	0.267	0.974	0.964	−0.819	0.177	−0.162	−0.163	0.921	1.000

**Table 6:** Results of correlation analysis of rainfall system parameters in the Yangtze River Delta region.

	${P}_{RCP}$	${T}_{EMP}$	${D}_{EWP}$	${S}_{LP}$	${S}_{TP}$	${W}_{DSP}$	${M}_{XSPD}$	${M}_{AX}$	${M}_{IN}$
${P}_{RCP}$	1.000	0.219	0.145	−0.255	0.161	0.066	0.088	0.129	0.178
${T}_{EMP}$	0.219	1.000	0.968	−0.859	0.398	−0.119	−0.083	0.973	0.977
${D}_{EWP}$	0.145	0.968	1.000	−0.866	0.408	−0.112	−0.083	0.922	0.975
${S}_{LP}$	−0.255	−0.859	−0.866	1.000	−0.508	0.067	0.021	−0.834	−0.849
${S}_{TP}$	0.161	0.398	0.408	−0.508	1.000	0.050	0.077	0.393	0.402
${W}_{DSP}$	0.066	−0.119	−0.112	0.067	0.050	1.000	0.923	−0.139	−0.087
${M}_{XSPD}$	0.088	−0.083	−0.083	0.021	0.077	0.923	1.000	−0.091	−0.065
${M}_{AX}$	0.129	0.973	0.922	−0.834	0.393	−0.139	−0.091	1.000	0.928
${M}_{IN}$	0.178	0.977	0.975	−0.849	0.402	−0.087	−0.065	0.928	1.000

## Multiple linear regression

This study utilizes the converted data from the three demonstration areas as respective samples. Rainfall ${P}_{RCP}$ is taken as the dependent variable, while dew point temperature ${D}_{EWP}$ or mean temperature ${T}_{EMP}$, sea level pressure ${S}_{LP}$, and mean wind speed ${W}_{DSP}$ or maximum sustained wind speed ${M}_{XSPD}$ are employed as the independent variables to carry out the multivariate linear regression statistical analysis and to establish the prediction models. The results of the regression coefficients and significance tests are shown in Table 7. All variables in the three regions exhibit significance levels below 0.05, passing the significance test and demonstrating statistical significance. In the Sichuan–Chongqing region, the unstandardized regression coefficients for ${D}_{EWP}$, ${S}_{LP}$, and ${W}_{DSP}$ are 0.019 > 0, −0.013 < 0, and 0.087 > 0, respectively, indicating a positive correlation between rainfall and ${D}_{EWP}$ and ${W}_{DSP}$, and a negative correlation with ${S}_{LP}$. In the Beijing-Tianjin-Hebei-Shandong region, the unstandardized regression coefficients for ${D}_{EWP}$, ${S}_{LP}$, and ${W}_{DSP}$ are 0.020 > 0, −0.003 < 0, 0.029 > 0, respectively, indicating that greater ${D}_{EWP}$ and ${W}_{DSP}$ lead to increased rainfall, while higher ${S}_{LP}$ results in reduced rainfall. In the Yangtze River Delta region, the unstandardized regression coefficients for ${T}_{EMP}$, ${S}_{LP}$, and ${M}_{XSPD}$ are −0.026 < 0, −0.075 < 0, and 0.018 > 0, respectively, which indicates that rainfall decreases as the ${T}_{EMP}$ and ${S}_{LP}$ increase and it rises as the ${M}_{XSPD}$ increases.

**Table 7:** Regression coefficients and significance tests.

Region	parameter	Unstandardized regression coefficient	Standard error	Standardized regression coefficient	Test results t	Significance	Tolerance	Variance expansion factor VIF
Sichuan–Chongqing	Constant	11.865	0.812		14.611	0.000		
	${D}_{EWP}$	0.019	0.001	0.227	35.544	0.000	0.533	1.876
	${S}_{LP}$	−0.013	0.001	−0.109	−17.094	0.000	0.530	1.887
	${W}_{DSP}$	0.087	0.003	0.125	25.732	0.000	0.921	1.085
Beijing–Tianjin–Hebei–Shandong	Constant	2.062	1.139		1.811	0.070		
	${D}_{EWP}$	0.020	0.001	0.302	35.548	0.000	0.344	2.909
	${S}_{LP}$	−0.003	0.001	−0.026	−3.092	0.002	0.354	2.824
	${W}_{DSP}$	0.029	0.002	0.081	15.920	0.000	0.951	1.051
Yangtze River Delta	Constant	77.785	1.128		68.979	0.000		
	${T}_{EMP}$	−0.026	0.001	−0.289	−41.017	0.000	0.258	3.879
	${S}_{LP}$	−0.075	0.001	−0.490	−69.804	0.000	0.260	3.853
	${M}_{XSPD}$	0.018	0.001	0.072	19.870	0.000	0.984	1.017

In summary, the multiple linear regression model for the Sichuan–Chongqing region is:

6 $${P}_{RCP}=11.865+0.019{D}_{EWP}-0.013{S}_{LP}+0.087{W}_{DSP}$$

The multiple linear regression model for the Beijing-Tianjin-Hebei-Shandong region is:

7 $${P}_{RCP}=2.062+0.020{D}_{EWP}-0.003{S}_{LP}+0.029{W}_{DSP}$$

The multiple linear regression model for the Yangtze River Delta region is:

8 $${P}_{RCP}=77.785-0.026{T}_{EMP}-0.075{S}_{LP}+0.018{M}_{XSPD}$$

## Testing and application

### Model performance testing

This paper employs various statistical methods, including the F-test, goodness-of-fit test, D-W test, covariance test and so on, to assess the established prediction model, as listed in Table 8, with the results displayed in Table 9.

**Table 8:** Equations of various testing methods.

Test	F-test	Goodness-of-fit test	D–W test	Covariance test
Equation	$F=\frac{SSR/k}{SSE/\left(n-k-1\right)}$ SSR is the square sum from regression, SSE is the square sum from residuals, and k is the degree of freedom	${R}^{2}=1-\frac{SSE}{SST}$ SSE is the square sum from residuals, SST is the total square sum	$D=\frac{\sum {\left({u}_{i}-{u}_{i-1}\right)}^{2}}{\sum {{u}_{i}}^{2}}$ D is the Durbin–Watson statistic, ${u}_{i}$ is the value of the ith residual in the residual sequence	$VIF=\frac{1}{T}$ VIF is the variance expansion factor, $T$ is the value of tolerance

**Table 9:** Model summary and overall significance test.

Region	*R*	${R}^{2}$	Standard error of estimate	Durbin–Watson value	Model	Square sum	Degrees of freedom	Mean square	F	Significance
Sichuan–Chongqing	0.331	0.109	1.087	1.677	Regression	5949.24	3	1983.08	1678	0.000
					Residual	48,456.47	40,994	1.18		
					Total	54,405.72	40,997			
Beijing–Tianjin–Hebei–Shandong	0.318	0.101	1.196	1.775	Regression	5818.84	3	1939.61	1355	0.000
					Residual	51,773.87	36,164	1.432		
					Total	57,592.71	36,167			
Yangtze River Delta	0.295	0.087	1.312	1.680	Regression	11,754.48	3	3918.16	2275	0.000
					Residual	122,861.39	71,336	1.722		
					Total	134,615.87	71,339			

The overall significance of the model is analyzed through the F-test, and the joint influence of all explanatory variables on the explained variables is tested to determine whether the multiple linear regression equation is established. As shown in Table 9, the F-test significance level is below 0.05 in all three regions, indicating that none of the independent variables in each region significantly affect rainfall, with a probability of 0. Moreover, there is a linear relationship between the dependent variable and the independent variable, resulting in the formulation of a multiple linear regression equation.

The determinable coefficient ${R}^{2}$ measures the goodness of fit between the data and the regression equation and serves as an important indicator of the relationship that exists between the explanatory variables and the explanatory variables. The value of the determinable coefficient is in the range of [0, 1], with values closer to 1 indicating a better fit. Table 9 reveals relatively small ${R}^{2}$ values for the multivariate linear regression equations in the three regions of Sichuan–Chongqing, Beijing-Tianjin-Hebei-Shandong and Yangtze River Delta, specifically 0.109, 0.101, and 0.087, respectively. These values indicate a general goodness of fit, signifying that rainfall possesses a certain linear correlation with ${D}_{EWP}$/${T}_{EMP}$, ${S}_{LP}$, and ${W}_{DSP}$/${M}_{XSPD}$.

The correlation between the variables is analyzed by using the D-W test (Durbin-Watson test), with results in Table 9 indicating values between 1.5 and 2.5, close to 2, demonstrating independence and no autocorrelation among the selected independent variables in each region. The model is constructed favourably, proving the validity of the first hypothesis of the multiple linear regression model.

The covariance test is a method of determining the presence of multicollinearity by examining the extent to which a given explanatory variable is explained by all other explanatory variables in the equation. Each explanatory variable in the equation has a variance expansion factor VIF, which is used to test whether covariance exists between independent variables. As shown in Table 7, the tolerances of each variable in the three regions are all greater than 0.1, and the VIF values are all less than 5, which indicates that there is no multicollinearity and extremely strong correlation link among the three independent variables selected in each region, confirming the validity of the second hypothesis of the multiple linear regression model.

### Residual distribution state test

**Figure 7:**
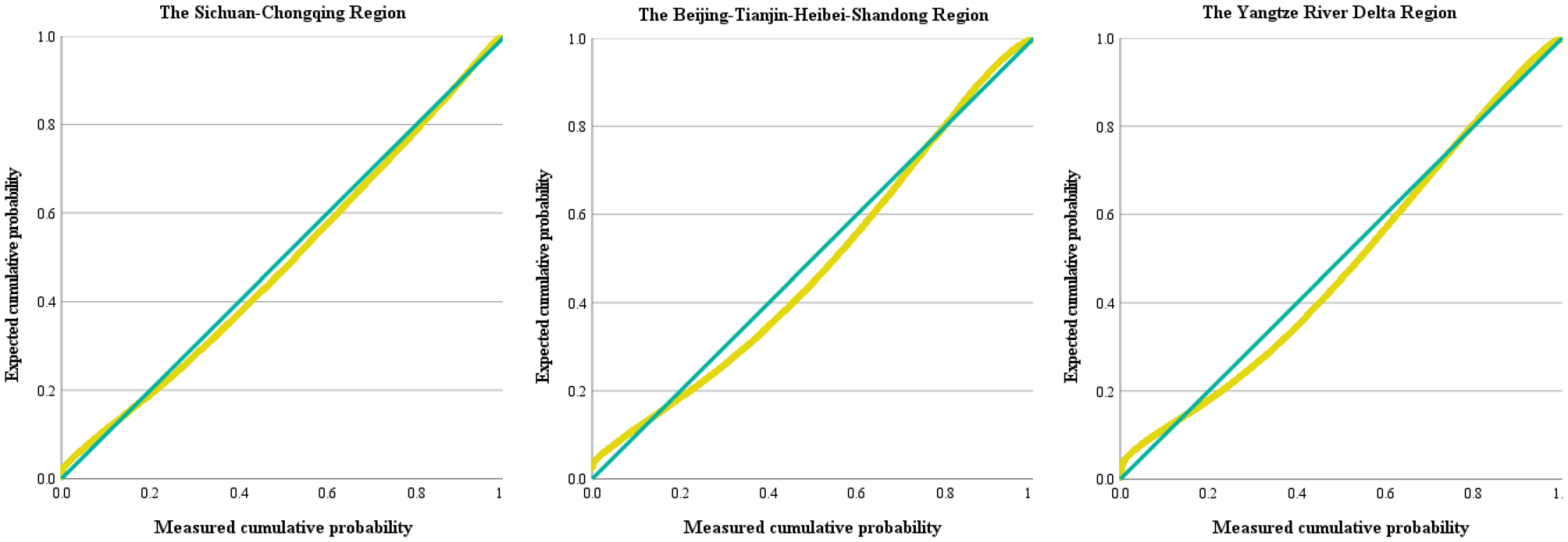
Probability–Probability plots of standardized residuals in three regions.

Probability–Probability plots are used to test the normality of data distribution, with simple linear regression requiring that regression residuals closely approximate a normal distribution. Figure 7 displays the Probability–Probability plots of the standardized residuals in the three demonstration districts, featuring a green diagonal line as the asymptote and a yellow curve representing the data distribution. The sample data primarily cluster around and closely align with the asymptote line, indicating that the residuals in the three districts conform to a normal distribution, affirming the validity of the third hypothesis of the multiple linear regression model.

**Figure 8:**
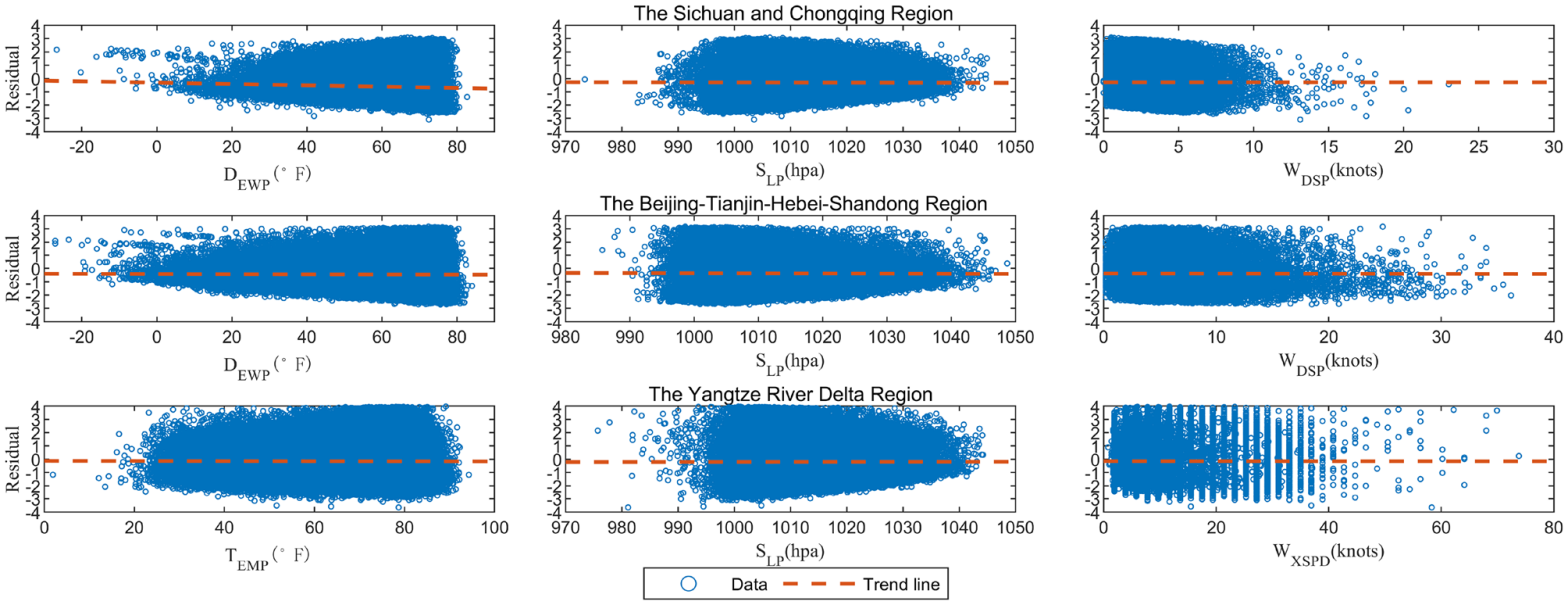
Distribution of residuals against various parameters residuals in three regions.

In order to further evaluate the effectiveness of the model parameter prediction equation, test the independence between the residual series and determine the fitting effect of the regression equation, residual analysis is usually used for diagnosis. The main element of residual analysis is to observe whether the overall trend of the distribution of residuals under different variables lies around y = 0. Taking the independent variables of the regression equations for the three regions as the horizontal coordinates and the residual values as the vertical coordinates, we plotted the corresponding residual distributions. As shown in Fig. 8, the fitted trend lines are basically close to or coincide with y = 0, and the residuals are basically distributed around the value of 0 throughout the entire stage, with no obvious upward or downward tendency, indicating that the regression model is well-fitted to the data. In addition, most of the residuals are uniformly concentrated in the [−2, 2] interval without linear changes, which further illustrates that there is no serial correlation between the residuals and good independence.

### Prediction for three regions

The constructed rainfall prediction models based on the theory of multiple linear regression are individually applied to the three demonstration areas to predict their effective rainfall data in 2022 reasonably. According to the National Standard of the People's Republic of China, “Standard for hydrological information and hydrological forecasting” (GB/T22482-2008)^44^, the accuracy of the medium and long-term rainfall forecasting adopts 20% of the multi-year measured variability as the evaluation standard. The forecasts smaller than the evaluation standard are designated as qualified forecasts, and the qualification rate is defined as the ratio of qualified forecast times to the total forecast times, as shown in Eq. (9). The results reveal that the Yangtze River Delta region boasts the highest forecast qualification rate at 77.8%, followed by the Sichuan–Chongqing region with a forecast qualification rate of 61.7%, while the Beijing–Tianjin–Hebei–Shandong region reports a forecast qualification rate of 58.6%, reaching the lowest. The qualification rate in the three areas is more than 50%, which is favorable and indicates that the prediction model has a certain degree of guiding significance in predicting rainfall for the three demonstration zones.

9 $$QR=\frac{n}{m}\times 100\%$$

where QR is the qualified rate (take one decimal), %; m is the number of qualified forecasts; m is the total number of forecasts.

## Conclusions

In this paper, the rainfall prediction models are constructed by using the rainfall meteorological data from a total of 94 meteorological stations in the Sichuan–Chongqing, Beijing-Tianjin-Heibei-Shandong and Yangtze River Delta regions from 1980 to 2021. Furthermore, the temporal distribution characteristics of rainfall towards transportation infrastructure in the past 42 years are analyzed. The main conclusions include:

1. The raw rainfall data in the Sichuan–Chongqing, Beijing-Tianjin-Hebei-Shandong, and Yangtze River Delta demonstration areas all exhibit conformity to the single-sided exponential distribution. There is a close alignment between the original data from the three regions and their respective designated probability distribution types, which lays the foundation for transforming the data into the standard normal space.
2. The input variables of the rainfall prediction model in the Sichuan–Chongqing region are determined as dew point temperature ${D}_{EWP}$, sea level pressure ${S}_{LP}$, and mean wind speed ${W}_{DSP}$. Similarly, the input variables of the remaining two regions can be determined as dew point temperature ${D}_{EWP}$, sea level pressure ${S}_{LP}$, and mean wind speed ${W}_{DSP}$ for the Beijing–Tianjin–Hebei–Shandong region, while mean temperature ${T}_{EMP}$, level pressure ${S}_{LP}$, maximum sustained wind speed ${M}_{XSPD}$ for the Yangtze River Delta region.
3. The Yangtze River Delta region has a higher risk of rainstorm disaster compared to the other two regions according to the frequency of rainfall and the return period of precipitation concentration. Notably, during July and August, the incidence of rainstorms and heavy rainstorm disasters in the Beijing–Tianjin–Hebei–Shandong region surpasses that in the Sichuan–Chongqing region. Furthermore, annual rainfall and rainstorm disasters display fluctuating upward and downward trends in the Sichuan–Chongqing region, respectively, from 1980 to 2021. Conversely, the Beijing–Tianjin–Hebei–Shandong and Yangtze River Delta regions experience a consistent upward trend in annual rainfall and rainstorm disasters during the same period.
4. Based on the multiple linear regression method, we have developed rainfall prediction models for the three demonstration areas. These models have successfully passed an overall significance test, demonstrating the independence of their variables, absence of multicollinearity, and normal distribution of residuals, signifying a certain level of effectiveness and reliability. Additionally, when predicting rainfall for 2022, the qualified prediction rate reaches 61.7%, 58.6%, and 77.8%, respectively, indicating favorable prediction results with significant guidance.

As the collection and collation of historical disaster information involves different levels in different departments, such as meteorology and water conservancy, coupled with a few missing measurements at some monitoring stations, discrepancies in results are not uncommon. This article takes the Sichuan–Chongqing, Beijing-Tianjin-Hebei-Shandong and Yangtze River Delta regions as typical demonstration areas, and mainly constructs the corresponding rainfall prediction models using multiple linear regression based on the rainfall meteorological data collected and collated by the wheat software. It also analyzes the temporal characteristics of rainfall oriented the resilience assessment of transportation infrastructure from 1980 to 2021, which is conducive to further understanding the trends over time of rainfall in the three regions. To a certain extent, it provides a reference for predicting future rainfall trends and assessing the resilience of transportation infrastructure. Nonetheless, due to data limitations, the rainfall analysis remains less comprehensive, and the underlying factors contributing to these patterns are not thoroughly explored. Hence, more systematic and in-depth studies will need to be conducted in the future.

## Data Availability

The datasets used and/or analyzed during the current study are available from the corresponding author upon reasonable request.
